# Simulation of three intraoral radiographic techniques in pediatric dental patients: subjective comfort assessment using the VAS and Wong-Baker FACES Pain Raiting Scale

**DOI:** 10.1186/s12903-020-1011-2

**Published:** 2020-01-31

**Authors:** Serife Ozdemir, Aysenur Parlakyıldız Gokce, Tugba Unver

**Affiliations:** 1 Department of Pediatric Dentistry, Bezmialem University Faculty of Dentistry, Adnan Menderes Bulvarı, Vatan Caddesi, 34093 Istanbul, Turkey; 2 Department of Pediatric Dentistry, Marmara University Faculty of Dentistry, Istanbul, Turkey; 3 Department of Oral and Maxillofacial Radiology, Bezmialem University Faculty of Dentistry, Istanbul, Turkey

**Keywords:** Radiography, Dental, Pediatric patient, Comfort assessment

## Abstract

**Background:**

Perception of pain associated with intraoral radiography in pediatric patients was evaluated through statistical comparisons of data obtained using the Wong-Baker FACES Pain Raiting Scale (WBFPRS) and visual analog scale (VAS) scoring.

**Methods:**

A total of 75 pediatric patients aged 6–12 years were included in this study. Simulations of each of three radiological methods (analog films, CCD sensor and phosphorus plates) were performed on 25 pediatric patients. Following the simulations, the meaning of each facial expression on the WBFPRS and the numbers on the VAS were explained to each child. For the comparison between groups, the homogeneity of the variances was tested with Levene’s test; because the variances were not homogeneous, Welch’s test was used. Tamhane’s T2 test was used because the homogeneity assumption was not provided to determine the source of the difference between the groups.

**Results:**

When the conventional method was compared to the PSPL (photostimulable phosphor luminescence) method, no significant differences were noted in either the WBFPRS or VAS results (p >0.05). The results obtained from both of the scales were significantly different between the conventional method and the CCD sensor method (*p* < 0.05). When the PSPL and CCD sensors were compared, a significant difference was observed for the WBFPRS (*p* < 0.05). It was found the highest level of pain scores when used the CCD sensor method than the analog film and PSPL methods (*p* < 0.05).

**Conclusions:**

It is expected that digital radiographic techniques will be improved in the future and that their disadvantages will be eliminated, resulting in imaging devices that are more comfortable for pediatric patients.

## Background

While early enamel lesions are likely to progress to the dentine, this process occurs relatively slowly over a period of at least 2 years. Early diagnosis of such enamel lesions allows timely intervention and helps to stop or reverse the progression of lesions [1]. Dental radiography is a very important diagnostic tool for the intraoral evaluation of children. In most cases, radiological investigations reveal important additional findings; however, the risks associated with radiography cannot be overlooked. The radiation dose should be kept as low as possible for both the patient and the dentist. Even if the radiation dose is very low, it is important to consider that radiation has the potential to cause biological harm. The younger the individual is, the greater the sensitivity to radiation due to the large number of dividing cells in young children. The motivations in obtaining radiographic images of the teeth and surrounding tissues in children mainly include the diagnosis of 1) decay, 2) traumatic dental injuries, 3) tooth eruption disorders, and 4) pathologies other than decay. Currently, digital radiographic methods are preferred over analog films in most clinics. The image quality achieved with digital radiography is similar to that of analog films but still depends on the digital system used. In terms of patient comfort, it has been reported that pediatric patients may find the abovementioned methods uncomfortable [2]. Although the use of these methods is quite common in dentistry, only a limited number of studies in the literature have reported the evaluation of patient comfort during such procedures [3]. There have been even fewer studies evaluating the comfort of pediatric patients during radiographic investigations [4–6]. Radiographic examination is usually considered a difficult, uncomfortable procedure for pediatric patients because the film is not easily positioned in the patients’ small mouths. Moreover, in some cases, the patients are less tolerant, more anxious and not as understanding [4]. Accordingly, although intraoral periapical radiographic methods are used in clinical practice, the preferred method should be the one associated with the least patient discomfort [7]. Two different imaging modalities can be used while obtaining X-rays – digital and conventional imaging [2]. Contrary to conventional imaging systems, digital imaging does not require the use of film bath solutions but instead involves the use of sensors that simultaneously form an image on a computer screen with the aid of computer-based imaging systems [8]. The image can be seen on a monitor within 0.5–120 s, which is significantly shorter than the time required for the conventional film bath process [9]. Direct digital imaging involves an X-ray device, an intraoral sensor and a computer [3, 9]. The sensors can be cabled or wireless. CCDs are the most commonly used image receptors in dental digital imaging and involve a semiconductive layer on a silicon chip that is sensitive to light and X-rays [8]. A PSPL (photostimulable phosphor luminescence) system is a wireless sensor consisting of a phosphorus-coated plastic plate that is not attached to a computer by a cable and is sensitive to X-rays [10]. The active area of phosphorus plates are larger than that of a CCD, and manipulating the former in the oral cavity is easier as they are thinner and more similar to periapical films in terms of size [11]. The outer surface of the sensors is generally rigid; therefore, placing them into the mouth to image the posterior region may be quite challenging, particularly in children. The sensors are fixed within the mouth either by pressure applied by the patient’s finger or by the use of sensor holders, after which the dentist makes necessary adjustments to the angle using a conventional radiographic device and initiates the exposure [12]. Today, the use of digital radiography systems is quite common, although some clinics still use the conventional method [2].

After a patient is prepared for an intraoral radiographic scan, the exposure is adjusted, the oral cavity is examined, the film and roentgen tube are positioned, and finally, the radiograph is obtained [8]. The child’s age, developmental status, cognitive and communication skills, and previously experienced pain should be considered when evaluating pain in pediatric patients. Obtaining accurate and reliable measurements of the level of pain in children is difficult, which has led to the development of several pain measurement methods for use in neonates, infants and children. Healthcare professionals should be able to understand the signs and symptoms of pain in children of different age groups, to identify whether the symptoms are due to pain or other factors in different groups [13] and to minimize pain and anxiety as much as possible while ensuring patient safety [12].

Children tend to become more capable of describing increases in pain with age and experience [14]. While selecting a method for the measurement of pain, factors such as the stage of pain development and the patient’s age and level of understanding should be considered, as well as functional status, abilities and emotional status [15]. By the time they reach the age of four, the majority of children are usually able to differentiate pain on a scale of 4–5. However, their ability to recognize pain develops as they become able to comprehend the intensity of pain, and this ability is usually developed at around the age of five [16]. To obtain pain reports in this age group, facial expression scales are generally used, in which children choose the facial expression that best describes the pain they feel or experience [17]. The scale developed by Wong and Baker is recommended for use in children aged 3 years and older. This scale requires health professionals to describe each face to the child, after which the child is asked to select the face that best reflects their current pain level. The pain score is determined based on the numerical values assigned to the faces, with the lowest and highest scores being 0 and 5, respectively; the high scores indicate lower pain tolerance, and the low scores reflect more tolerable pain [16]. Upon reaching the age of 7–8 years, children begin to understand the quality of pain. The VAS (visual analog scale) is the most commonly preferred method for use in this age interval [16].

The present study evaluates perception of pain associated with intraoral radiography in pediatric patients through statistical comparisons of data obtained from the Wong-Baker FACES Pain Raiting Scale (WBFPRS) and VAS scoring.

## Methods

Approval for this study (71306642) was obtained from the Bezmialem Vakif University Rectorate Clinical Research Ethics Committee. A total of 75 pediatric patients aged 6–12 and their parents, who were referred to Bezmialem Vakif University Department of Pediatric Dentistry for examination and had no systemic disease, physical disability or intellectual disability, pain, acute infection or trauma were included in the study. The parents of the included children were given a detailed explanation of the purpose and methods of the planned clinical research study and provided verbal and written informed consent for participation in the study.

Power analysis was performed, and the results of the power calculation considering the VAS variance revealed that the minimum sample size for each group was 25. This value was determined by projecting the power as 0.80, the mean difference as △ = 1, and the significance level as a = 0.05.

**Fig. 1:**
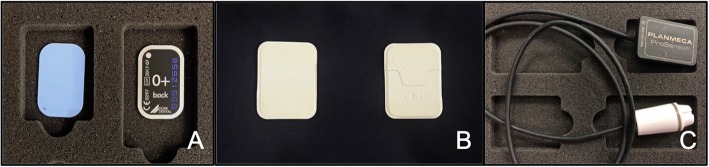
Sensors used in this study, from left to right: (**a**) DÜRR Dental PSPL sensor (Bietigheim-Bissingen, Germany), (**b**) CEA intraoral film (Strängnäs, Sweden) and (**c**) Planmeca® Prosensor (Helsinki, Finland)

In this study, three different intraoral radiographic methods were used: analog films (CEA intraoral film, Sweden), and CCD sensors (Planmeca® Prosensor, Finland) and phosphorus plates (DÜRR Dental PSPL sensor, Germany), with the latter two as the digital methods (Fig. 1).

Simulations of each of these three radiological methods were performed on 25 pediatric patients. To avoid for formation of potential age clusters, these participants were randomly divided into simulation groups with equal numbers of patients aged 6–12. Because wearing lead aprons and thyroid protectives may also affect patient comfort, these items were avoided to ensure that the results were not affected during the simulations.

Each simulation was performed at the same site for each participant by positioning the sensors to obtain periapical films of the right posterior teeth of the lower jaw and applying a bisector technique. To avoid any cross-infection, the sensors were placed in single-use plastic sheaths before each simulation. All scan simulations were performed by the same individual who used the same-sized films and sensors to ensure standardization. Size 0 films and sensors, which are appropriate for pediatric patients, were preferred in this study; the size of the CCD sensor was 33.6 × 23.4 × 14 mm, that of the analog film was 22 × 35 × 0.77 mm, and that of the PSPL sensor was 21 × 31 × 1 mm.

In parallel to the exposure time, a simulation time of 10 s was selected.

**Fig. 2:**
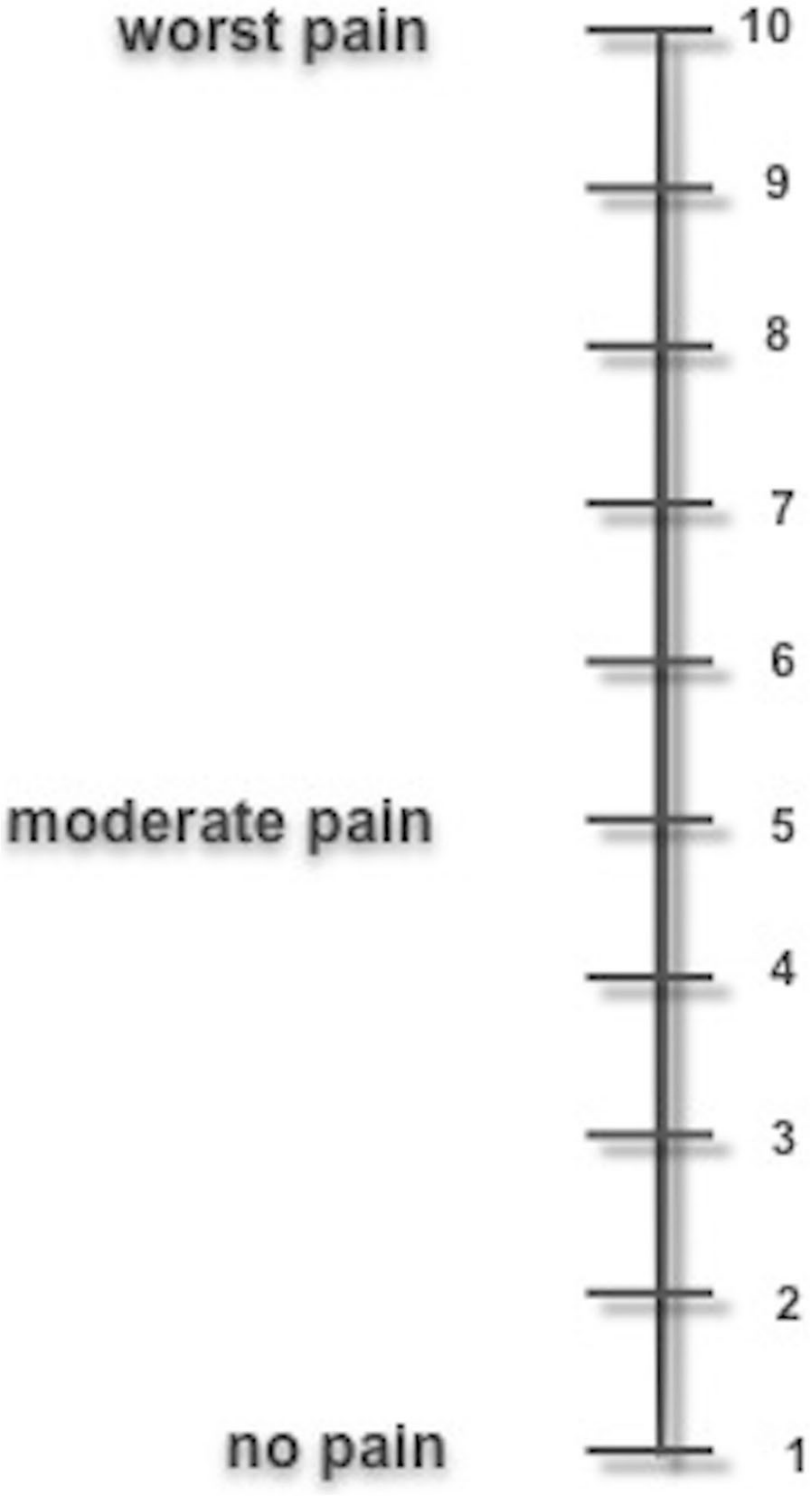
VAS used for the study

**Fig. 3:**
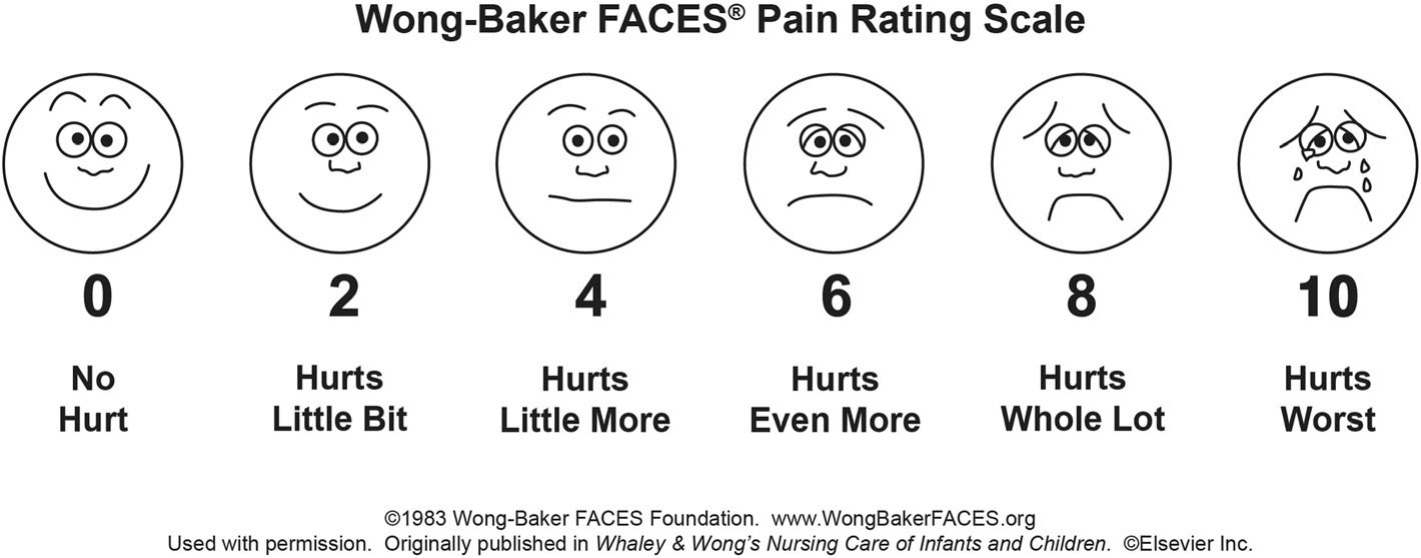
WBFPRS used for the study

Following the simulation, the meaning of each facial expression according the WBFPRS and the numbers on the VAS (Fig. 2) were explained to each child, and the children were then asked to evaluate the time period of the simulation in terms of comfort and to mark the values they deemed appropriate on the WBFPRS (Fig. 3) and the VAS. Because the vertical or horizontal alignment of the VAS does not affect the results, the scale was positioned vertically to facilitate the children’s understanding of the VAS. The obtained comfort scores were statistically compared based on the receptor type used. The required permission for publishing the use of the Wong-Baker FACES Pain Raiting Scale was received from the Wong–Baker FACES Foundation.

Each child was radiographed with only one imaging technique, and the pain perception of the patients against the radiographic technique was evaluated with two different scales.

The statistical analyses were performed using SPSS 24.0 (Statistical Package for the Social Sciences) program. For the comparison between groups, the homogeneity of the variances was tested with Levene’s test; because the variances were not homogeneous, Welch’s test was used. Tamhane’s T2 test was used because the homogeneity assumption was not provided to determine the source of difference between the groups. The 0.05 significance level was used to test the significant differences between the average points of the groups.

## Results

A total of 75 patients were included in the study, and the median age was 9 years. There was no statistically significant difference between the ages of the patients in the radiography method groups [FWelch = .107, *p* > 0.05]. A total of 53.3% of the patients were females, and 46.7% were males. When patient comfort was considered, in the study groups, 64% of the female (F) participants were examined using the analog film, 60% using CCD, and 36% using PSPL. 64% of the male (M) participants were examined using the PSPL, 40% using CCD, and 36% using analog films.

The gender distribution was homogeneous in all groups, and there was no significant difference between male and female participants when comfort was compared using the three methods. The descriptive statistics of age, WBFPRS and VAS by subgroups and those for the total sample are shown in Table 1.

**Table 1:** Descriptive statistics of age, WBFPRS and VAS by subgroups and for the total sample

Group		AGE	^a^WBFPRS	^b^VAS
ANALOG FILM (*n* = 25)	Median	9.00	1.00	0.00
	Minimum	6	0	0
	Maximum	12	2	3
	Mean	8.96	.72	.76
	Std. Deviation	2.010	.737	1.012
^c^PSPL (*n* = 25)	Median	9.00	0.00	1.00
	Minimum	6	0	0
	Maximum	12	2	3
	Mean	8.96	.44	.92
	Std. Deviation	1.989	.583	.997
^d^CCD SENSOR (*n* = 25)	Median	9.00	2.00	1.00
	Minimum	6	0	0
	Maximum	12	3	4
	Mean	8.76	1.40	1.72
	Std. Deviation	1.615	1.041	1.487
TOTAL (*n* = 75)	Median	9.00	1.00	1.00
	Minimum	6	0	0
	Maximum	12	3	4
	Mean	8.89	.85	1.13
	Std. Deviation	1.857	.896	1.245

^a^*WBFPRS* Wong-Baker FACES Pain Raiting Scale

^b^*VAS* Visual Analog Scale

^c^*PSPL* Photostimulable Phosphor Luminescence

^d^*CCD* Charge-Coupled Device

The WBFPRS pain scores obtained using the radiographic methods were initially compared by checking the homogeneity of variance by Levene’s test (F (2.72) = 7.535, *p* < 0.05), and the methods were compared using Welch’s ANOVA test. According to the WBFPRS pain scores, there was a statistically significant difference between the radiographic method groups (FWelch = 8.017, *p* < 0.05) (Table 2). When pain was examined using the CCD sensor method according to conventional and PSPL methods, there was a statistically significant difference between the scores (*p* < 0.05). The average differences showed that patients who were examined using the CCD sensor method had higher pain scores than those of patients examined using the analog film and PSPL methods.

**Table 2:** Post hoc comparisons with Tamhane’s T2 test among the radiography groups according to the ^*^WBFPRS

(I) Radiography Groups	(J) Radiography Groups	Mean Difference (I-J)	Std. Error	Sig.	95% Confidence Interval	
					Lower Bound	Upper Bound
Conventional Method	PSPL	.280	.187	.371	1859	.745
	CCD Sensor	- 680^*^	.255	.032	−1.3136	.046
^**^PSPL	Conventional Method	−.280	.187	.371	−.7459	.185
	CCD Sensor	−.960^*^	.238	.001	−1.5561	−.363
^***^CCD Sensor	Conventional Method	.680^*^	.255	.032	.0464	1.313
	PSPL	.960^*^	.238	.001	.3639	1.556

*P < 0.05

^*^WBFPRS Wong-Baker FACES Pain Raiting Scale

^**^PSPL Photostimulable Phosphor Luminescence

^***^CCD Charge-Coupled Device

To determine whether the VAS scores differed significantly according to the radiographic method groups, homogeneity of variance was checked by Levene’s test (F (2.72) = 5.726, *p* < 0.05), followed by Welch’s test. According to the VAS pain scores, there is a statistically significant difference between the radiographic method groups (FWelch = 3.664, *p* < 0.05). Tamhane’s T2 test was applied to determine which groups contributed to the difference (Table 3). When pain was examined using the CCD sensor method according to the conventional method pain scores, there was a statistically significant difference between the scores (*p* < 0.05). The average differences showed that patients who were examined using the CCD sensor method had higher pain scores than those of patients examined using the traditional method.

**Table 3:** Post hoc comparisons with Tamhane’s T2 test among the radiography groups according to the ^*^VAS

(I) Radiography Groups	(J) Radiography Groups	Mean Difference (I-J)	Std. Error	Sig.	95% Confidence Interval	
					Lower Bound	Upper Bound
Conventional Method	PSPL	−.160	.284	.924	−.862	.542
	CCD Sensor	−.960^*^	.359	.032	−1.854	−.066
^**^PSPL	Conventional Method	.160	.284	.924	−.542	.862
	CCD Sensor	−.800	.357	.090	−1.690	.090
^***^CCD Sensor	Conventional Method	.960^*^	.359	.032	.066	1.854
	PSPL	.800	.357	.090	−.090	1.690

*P < 0.05

^*^VAS Visual Analog Scale

^**^PSPL Photostimulable Phosphor Luminescence

^***^CCD Charge-Coupled Device

The subjects with the CCD sensor complained more than did those examined using the conventional and PSPL methods. According to the results, the CCD sensor method was the most disruptive.

## Discussion

During conventional and digital radiological imaging studies, patient comfort may be negatively affected depending on the physical characteristics of the sensors used. Although patient comfort affects the dose of radiation to which patients are exposed, as radiographic procedures need to be repeated when the results are not satisfactory, this topic has been investigated in only a limited number of studies [3–6, 18]. The present study is unique in that no previous study has investigated patient comfort in pediatric patients in the 6–12 age group.

In a study carried out by Wenzel et al. [18], patient comfort during bitewing radiography obtained in 130 adult patients using either a CCD sensor or a PSPL sensor were compared and evaluated based on the VAS. The results of that study showed that the PSPL sensors were more comfortable than the CCD sensors for bitewing radiography. However, contrary to the present study, that study did not involve the use of conventional methods and compared only digital methods.

In another study, Gonçalves et al. [3] evaluated patient comfort in 300 adult subjects using the VAS during periapical radiographic simulations performed with a film holder and holder-free analog films, as well as with different CCD and PSPL sensors. The authors reported that the highest level of patient comfort was achieved with the PSPL sensor, followed by the CCD sensors, film holder-free analog films, and film holder analog films. According to these researchers, the reasons for these results were the thickness, flexibility and round corners of the PSPL sensor. Although the PSPL sensor and analog film we used in our study received a score of 0, the CCD sensor received a score of 2. These scores may explain the lack of statistical significance between the PSPL sensor and analog film groups in our study. Additionally, it is thought these results are different from those of our study because of the mean age of the patient groups. In addition, we preferred not to use film holders in our research because they already contribute to reducing patient comfort, and we planned to compare digital sensors and analog films with only the discomfort caused by their physical properties.

Even though the image quality achieved with digital radiography is similar to that achieved with conventional radiography, the digital system used is still an important factor. No previous study has investigated the use of digital radiography in children, and the advantages of these methods are undervalued due to their inherent disadvantages, such as the acceptability of phosphorus plaques or sensors by children.

Dölekoğlu et al. [19] reported that digital imaging systems are not preferred by general dentists aged 40–50 because of the high costs associated with these systems and the general lack of knowledge of this technology. The major reasons for using digital radiography are the reduced radiation dose and the ability to edit the image if necessary for optimal display [19].

In general dental practice, the number of dentists who prefer using digital dental radiography continues to grow. In the past, the type and use of digital radiographic equipment has already been the subject of several studies in multiple countries. These studies have shown an increased availability of dental radiographic units throughout the years, with an exponential increase in digitalization. In a recent survey, 90% of the respondents in Belgium reported using digital intraoral radiography [20]. In 2005, Ilgüy et al. [21] reported that 14% of general dentists used digital radiography and that 6.5% used panoramic units. In total, 39.4% of dentists practicing in a hospital and 2.2% of dentists working in a private practice use panoramic units [21]. In a more recent survey conducted in 2011 by Dölekoğlu et al. [19], 67% of the respondents reported using digital radiography. The percentage of dentists using digital radiography in Turkey was higher than that in other countries [22–24].

The statistical analyses of the data collected in this study indicated that the level of patient comfort was not significantly different between the PSPL sensor and analog film, while the children reported the CCD sensor to be the most uncomfortable. When the radiographic techniques were compared, there was a significant difference between the analog film and the CCD sensor on both pain scales, whereas a significant difference was found between the PSPL and CCD sensors when compared on the WBFPRS only. According to a study on validating the VAS and WBFPRS pain scales in children, the WBFPRS has the potential to be an excellent measure of treatment effects in school-age children and adolescents [25]. The results of our current study suggest that children have more difficulty using the VAS than the WBFPRS.

Considering these results, we believe that the thickness of the CCD sensor constitutes a great disadvantage. However, there was no statistically significant difference between the PSPL sensor and analog film because of their similar size. All three methods were applied without using film holders; the film/sensor was placed in the mouth parallel to the long axis of the tooth and was held in the mouth by the patient’s finger. We considered the influence of the oral characteristics, such as mouth opening and palate width of the subjects, to be negligible because the randomization of the subjects yielded no significant differences in baseline oral health status and oral cavity structures between the groups. Because the simulation was performed on pediatric patients without using a film holder, we believe that comfort was more negatively affected, especially in the CCD sensor group, because of the rigidity of the sensor and the difficulty of stabilizing it in the mouth. Based on these findings, we suggest that the thickness and size of the sensor and the involvement of a cable are among the most important factors affecting patient comfort.

## Conclusions

According to the results of this study, the CCD sensor causes the most disturbance, and there is no significant difference in discomfort between using the PSPL method and using the analog film.

Nevertheless, as conventional methods involve the use of bath solutions, prohibit any image adjustment and result in the loss of time, the use of PSPL as a digital method may be recommended for children. The patient-reported level of discomfort for this sensor appears to be mild, and the use of this sensor is associated with certain advantages, such as saving time, allowing adjustments to be made, reducing the radiation dose, and permitting measurements to be made on the images while also eliminating the need for chemical procedures and consequently minimizing environmental pollution. However, large-scale studies are required to evaluate the comfort associated with different radiographic methods in pediatric patients.

## Data Availability

The data that support the findings of this study are available on request from the corresponding author. The data are not publicly available due to [restrictions, e.g., they contain information that could compromise the privacy of the research participants].

## Abbreviations

APPT: Adolescent Pediatric Pain Tool
CCD: Charge-Coupled Device
CFCS: Child Facial Coding System
CMOS/APS: Complementary Metal Oxide Semiconductor/Active Pixel Sensor
F: Female
M: Male
PSPL: Photostimulable Phosphor Luminescence
SPSS: Statistical Package for the Social Sciences
VAS: Visual Analog Scale
WBFPRS: Wong-Baker FACES Pain Raiting Scale

## References

[1] European Comission Guidelines on Education and Training in Radiation Protection for Medical Exposures 2018. Available at: https://ec.europa.eu/energy/sites/ener/files/documents/116.pdf. Accessed 1 Aug 2018.

[2] Espelid I, Mejare I, Weerheijm K (2003). EAPD guidelines for use of radiographs in children. Eur J Paediatr Dent.

[3] Goncalves A, Wiezel VG, Goncalves M, Hebling J, Sannomiya EK (2009). Patient comfort in periapical examination using digital receptors. Dentomaxillofac Radiol.

[4] da Silva Pierro VS, Barcelos R, de Souza IPR (2008). Pediatric bitewing film holder: preschoolers’ acceptance and radiographs’ diagnostic quality. Pediatr Dent.

[5] Pitts NB, Hamood SS, Longbottom C (1990). An in-vivo evaluation in children of the HPL bitewing device. J Dent.

[6] Pitts NB, Hamood SS, Longbottom C, Rimmer PA (1991). The use of bitewing positioning devices in children's dentistry. Dentomaxillofac Radiol.

[7] American Academy of Pediatric Dentistry (2012). Guideline on prescribing dental radiographs for infants, children, adolescents, and persons with special health care needs. Endorsements.

[8] White SC, Pharoah MJ (2004). Oral Radiology Principles and Interpretation.

[9] Abesi F, Mirshekar A, Moudi E (2012). Diagnostic accuracy of digital and conventional radiography in the detection of non-cavitated approximal dental caries. Iran J Radiol.

[10] Whaites E, Drage N (2002). Essentials of Dental Radiography and Radiology Dental Panoramic Tomography.

[11] Aps J (2013). Three-dimensional imaging in paediatric dentistry: a must-have or you’re not up-to-date?. Eur Arch Paediatr Dent.

[12] Srouji Rasha, Ratnapalan Savithiri, Schneeweiss Suzan (2010). Pain in Children: Assessment and Nonpharmacological Management. International Journal of Pediatrics.

[13] Morton NS (1997). Pain assessment in children. Paediatr Anaesth.

[14] Abu-Saad HH, Hamers JP (1997). Decision-making and paediatric pain: a review. J Adv Nurs.

[15] Butterworth J, Mackey D, Wasnick J, Morgan G, Mikhail M, Morgan G (2013). Morgan and Mikhail’s Clinical Anesthesiology.

[16] Brand K, Thorpe B (2016). Pain assessment in children. Anaesth Intensive Care Med.

[17] von Baeyer CL (2006). Children’s self-reports of pain intensity: scale selection, limitations and interpretation. Pain Res Manag.

[18] Wenzel A, Frandsen E, Hintze H (1999). Patient discomfort and cross-infection control in bitewing examination with a storage phosphor plate and a CCD-based sensor. J Dent.

[19] Dölekoğlu S, Fişekçioğlu E, İlgüy M, İlgüy D (2011). The usage of digital radiography and cone beam computed tomography among Turkish dentists. Dentomaxillofac Radiol.

[20] Snel Robin, Van De Maele Ellen, Politis Constantinus, Jacobs Reinhilde (2018). Digital dental radiology in Belgium: a nationwide survey. Dentomaxillofacial Radiology.

[21] İlgüy D, İlgüy M, Dinçer S, Bayırlı G (2005). Survey of dental radiological practice in Turkey. Dentomaxillofac Radiol.

[22] Jacobs R, Vanderstappen M, Bogaerts R, Gijbels F (2004). Attitude of the Belgian dentist population towards radiation protection. Dentomaxillofac Radiol.

[23] Brian JN, Williamson GF (2007). Digital radiography in dentistry: a survey of Indiana dentists. Dentomaxillofac Radiol..

[24] Aps JKM (2010). Flemish general dental practitioners’ knowledge of dental radiology. Dentomaxillofac Radiol..

[25] Garra G, Singer AJ, Taira BR, Chohan J, Cardoz H, Chisena E, Thode HC (2010). Validation of the Wong-Baker FACES Pain Rating Scale in pediatric emergency department patients. Acad Emerg Med.

